# Supplementary material for: Splice-Junction-Based Mapping of Alternative Isoforms in the Human Proteome
Source: Cell Rep. Author manuscript; Available in PMC 2020 Jan 15. (PMC6961840; doi:10.1016/j.celrep.2019.11.026)
Supplement: 2 [file NIHMS1546469-supplement-2.zip › DF1/PXD006675/PulmonaryVein/PulmonaryVein_31_RILPL1_ASSDAAESALEK.pdf]

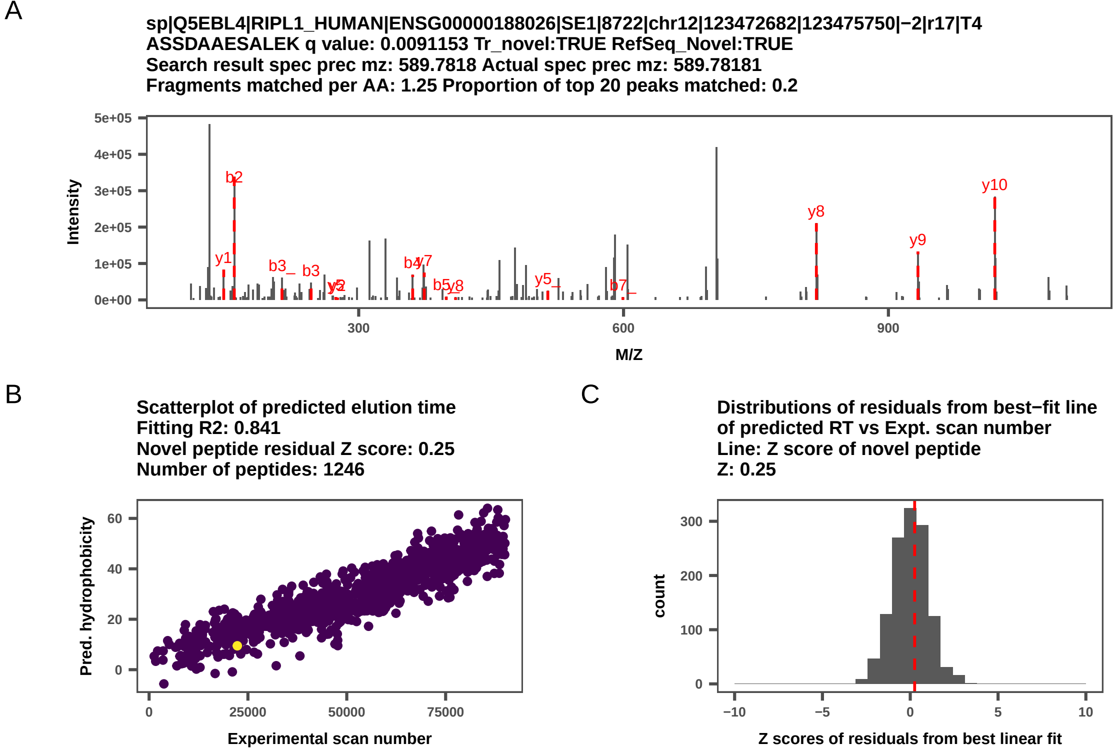
A
sp|Q5EBL4|RIPL1_HUMAN|ENSG00000188026|SE1|8722|chr12|123472682|123475750|−2|r17|T4
ASSDAAESALEK q value: 0.0091153 Tr_novel:TRUE RefSeq_Novel:TRUE
Search result spec prec mz: 589.7818 Actual spec prec mz: 589.78181
Fragments matched per AA: 1.25 Proportion of top 20 peaks matched: 0.2
Intensity
5e+05
4e+05
3e+05
2e+05
1e+05
0e+00
b2
y1
b3_
b3
y2
b4
y7
b5
y8
y5_
b7_
y8
y9
y10
300
600
900
M/Z
B
Scatterplot of predicted elution time
Fitting R2: 0.841
Novel peptide residual Z score: 0.25
Number of peptides: 1246
Pred. hydrophobicity
60
40
20
0
25000
50000
75000
Experimental scan number
C
Distributions of residuals from best−fit line
of predicted RT vs Expt. scan number
Line: Z score of novel peptide
Z: 0.25
count
300
200
100
−10
−5
5
10
Z scores of residuals from best linear fit
